# Comparison of the Cervex-Brush^®^ Combi and the Cytobrush+Ayres Spatula Combination for Cervical Sampling in Liquid-Based Cytology

**DOI:** 10.1371/journal.pone.0164077

**Published:** 2016-10-14

**Authors:** Marcelo Simonsen, José Humberto Tavares Guerreiro Fregnani, Júlio Cesar Possati Resende, Márcio Antoniazzi, Adhemar Longatto-Filho, Cristovam Scapulatempo-Neto

**Affiliations:** 1 Gynecology Department, Santa Casa de São Paulo, São Paulo, Brazil; 2 Research and Teaching Institute, Barretos Cancer Hospital, Barretos, Brazil; 3 Cancer Prevention Department, Barretos Cancer Hospital, Barretos, Brazil; 4 Molecular Oncology Center, Barretos Cancer Hospital, Barretos, São Paulo, Brazil; 5 Laboratory of Medical Investigation (LIM-14), School of Medicine, University of São Paulo, São Paulo, Brazil; 6 Pathology Department, Barretos Cancer Hospital, Barretos, São Paulo, Brazil; Universidade Estadual de Maringa, BRAZIL

## Abstract

**Objectives:**

To compare the performance of two cervical collection devices (Cytobrush+Ayres spatula and Cervex-Brush^®^ Combi) for cellular sampling, transformation zone representation and accuracy in diagnosing cervical intraepithelial neoplasia (CIN) 2+.

**Methods:**

Cervical samples were collected from patients referred to the colposcopy unit of the Barretos Cancer Hospital between September 2013 and October 2014 using one of the two sampling devices. Additionally, colposcopy was performed with or without cervical biopsy and/or endocervical curettage.

**Results:**

Biopsy was performed in 670 of the 1,235 patients submitted to colposcopy (54.2%). The Cervex-Brush^®^ Combi was more effective than the Cytobrush with respect to endocervical cells sampling (82.7% versus 74.6%; p = 0.001). Sensitivity was also higher with the Cervex-Brush^®^ Combi (48.6% versus 33.9%; p = 0.023) for predicting CIN2+ when high-grade squamous intraepithelial lesions were detected at cytology.

**Conclusions:**

Cervex-Brush^®^ Combi was more effective than Cytobrush+Ayres Spatula for endocervical cells sampling and also had a slightly higher accuracy in predicting histologically CIN2+ lesions in patients with diagnosis of HSIL in cytology.

## Introduction

Cervical cytology is considered an effective method for detecting cervical cancer and its precursors. After the implementation of the screening programs there was an important reduction in both incidence and mortality of cervical cancer[1–3]. In ideal conditions, specificity of this test is fairly satisfactory for the detection of precancerous lesions [4]. Although some countries have adopted human papillomavirus (HPV) testing as the test of choice for population-based screening, cervical cytology remains a fundamental step of the subsequent investigation [5].

In Brazil, the Ministry of Health recommends that all sexually active women between 25 and 64 years of age should perform Papanicolaou testing for cervical cancer screening [6]. The Brazilian government program covers the costs only of conventional cytology. Although a previous meta-analysis showed no advantages of liquid based cytology over conventional cytology [7], our group already published the superiority of liquid based cytology for detection of abnormalities in Papanicolaou testing. Other advantage of liquid based cytology is the use of remaining material for HPV testing and for cell preservation [8, 9].

Cervical sampling devices are believed to play an important role in the quality of the sample [10]. Around 60% of false-negative results are potentially associated with the type of device [11–13]: the capacity of collecting and releasing different type of cells varies according to the device`s shape and the material of what it`s made [9]. The ideal sampling device should also be adequately priced for population-based screening and be capable of collecting a sufficient amount of cells from the cervix and from the squamocolumnar junction, with minimum discomfort and mucosal injury [14, 15].

**Fig 1:**
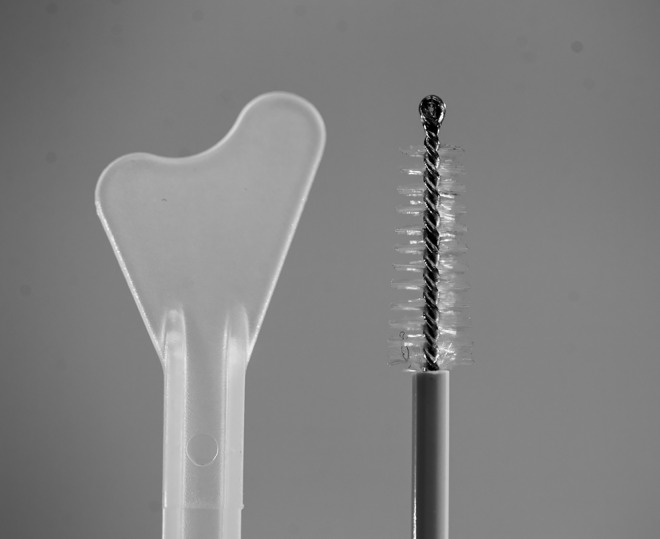
Cytobrush+Ayres spatula.

Various cervical sampling devices were evaluated in previous studies [10], with particular emphasis on the Cytobrush+Ayres spatula (Fig 1) combination and the Cervex-Brush^®^, which have been proven to be very effective for ectocervical and endocervical cells sampling [10, 16]. The Cytobrush has been shown to be more effective in collecting a greater percentage of endocervical cells [17] although some authors have reported little difference between the two methods [15, 18, 19].

**Fig 2:**
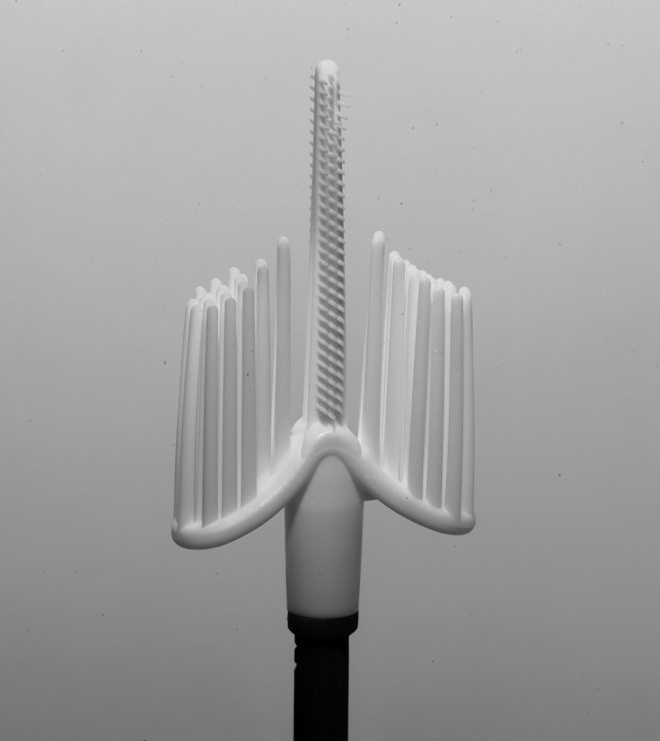
Cervex-Brush^®^ Combi.

The Cervex-Brush^®^ Combi (Fig 2) is a newer version of the Cervex-Brush^®^ with a longer central axis with tiny crossed bristles. A study comparing the use of these two devices in liquid-based cytology found them to be similar with respect to the number of squamous cells collected; however, Combi version collected 2–3 times more endocervical cells and was associated with a significantly greater number of abnormalities identified in the samples [20].

Few studies have been conducted to compare the use of different cervical sampling devices in liquid-base cytology [21] and, none of them has correlated the cytological and histological findings of patients whose samples were obtained using the Cervex-Brush^®^ Combi and the Cytobrush. The objective of the present study was to evaluate the accuracy of liquid-based cytology using the Cytobrush+Ayres spatula versus the Cervex-Brush^®^ Combi for the diagnosis of CIN 2+.

## Materials and Methods

### Research site and ethical aspects

This study was performed at the Barretos Cancer Hospital, Barretos, São Paulo, Brazil. The colposcopies and cervical cytologies were carried out at the Cancer Prevention Department, the slides were evaluated at the Pathology Department.

The internal review board of the Barretos Cancer Hospital approved the study in August 2013 under approval number 362.243. Explanations on the nature of the study were provided and all patients who agreed to participate signed an informed consent form.

### Population

The participants of this study were referred to the Barretos Cancer Hospital by other healthcare units or by the hospital’s own mobile screening unit between September 9^th^, 2013 and October 10^th^, 2014. All of them had abnormal cervical cytology in routine cervical sampling with any kind of positive finding (ASCUS+) and were reexamined at the hospital and submitted to colposcopy with or without biopsy. Cases in which colposcopy was satisfactory and the findings did not justify performing a biopsy were included in the study. Patients under 18 years of age were excluded, as well tests with cytology and biopsy evaluated on the same day by the same pathologist.

### Cytology sampling and colposcopy

Cervical cytology samples were collected immediately before the colposcopy alternately using the Cervex-Brush^®^ Combi or the Cytobrush (Rovers^®^ Medical Devices B.V., The Netherlands) with the Ayres spatula, i.e. on each day of the week, sampling was performed with one of the two devices. Material for cervical cytology was collected immediately prior to colposcopy. Two gynecologists from the Cancer Prevention Department (JCPR and MA) were responsible for performing both procedures.

The technique used to collect samples for cytopathology was carried out in accordance with the manufacturer’s instructions. For samples collected using the Cervex-Brush^®^ Combi, the brush was delicately inserted into the endocervical canal until the lateral bristles were touching the ectocervix. The brush was then rotated two full turns clockwise. With the Cytobrush+Ayres spatula, the spatula was rotated one complete turn clockwise and the Cytobrush was inserted gently into the endocervical canal and then rotated two full turns. Once disconnected from the handles, the brush heads were deposited into a SurePath^®^ vial containing liquid preservative.

For the study purposes, the cervical cytological was denominated as ASCUS+ (ASCUS, ASCH, LSIL, AGC, HSIL, in situ adenocarcinoma or invasive carcinoma), ASCH+ (ASCH, AGC, HSIL, in situ adenocarcinoma or invasive carcinoma) or HSIL+ (HSIL, in situ adenocarcinoma or invasive carcinoma). We didn’t evaluate in separate the LSIL+ group because the clinical relevance is similar to ASCUS+. In the same way, we included the subclasses AGC-not otherwise specified (NOS) and AGC- favor neoplastic in the group AGC because this discrimination doesn’t have clinical implications.

Colposcopy was conducted with prior knowledge of the referral cytology findings by two experienced gynecologists (J.C.P.R and M.A). Acetic acid 3% and Lugol’s solution were applied to the cervix and vaginal canal, with biopsy being performed if colposcopic findings were abnormal.

If the squamocolumnar junction was not visible at colposcopy and the referral cytology findings were of low-grade squamous intraepithelial lesions or higher (LSIL+), endocervical curettage was performed, with these results being considered the gold standard. In women whose colposcopy results were normal but whose referral cytology report was of high-grade squamous intraepithelial lesions (HSIL) or if there was a high-grade lesion for which it proved impossible to evaluate invasion, endocervical curettage was performed even when colposcopy was satisfactory. In the cases in which colposcopy was satisfactory, with no abnormal findings, and the referral cytology report was indicative of LSIL or less, neither biopsy nor endocervical curettage were performed.

When colposcopy revealed major findings but the biopsy indicated a normal cervix or chronic cervicitis, the histological findings from the sample obtained by loop excision were considered the gold standard.

### Preparation and analysis of the slides

The pathology laboratory received the SurePath^®^ vials, duly identified and containing the detachable brush heads from the devices as a criteria for sample acceptance and processing. The samples were processed according to manufacture´s instructions. The slides were confectioned and Papanicolaou stained using PrepStain^™^ slide processor and finally the slides were mounted with a coverslip.

Next, experienced cytology technicians analyzed the slides and registered the findings in the hospital’s database. This system automatically selected 10% of the negative slides to be reviewed by a pathologist as internal quality control. All unsatisfactory, suspected and positive cases were reviewed by pathologists from Barretos Cancer Hospital.

### Data collection and calculation of the sample size

Based on data from the institution, a retrospective data analysis showed a better correlation between cytology and histopathology when cytology was collected using Cytobrush, with this correlation being around 8% more accurate than cytologies collected with Combi-Brush^®^. Considering an alpha error of 5%, a power of the test of 80% and comparing the percentage of correct diagnoses, sample size was calculated at 391 patients in each study group.

When obtaining the cervical samples, the physician in charge recorded the type of used device for sampling and his/her own name on the data collection form. At the end of each month, all forms were completed with the cytopathology and biopsy/ curettage results, as well as the colposcopic findings. The completed forms were sent to Barretos Cancer Hospital Researchers Support Center to be organized into a databank for posterior statistical analysis.

### Statistical Analysis

The population was characterized using descriptive statistics: absolute frequencies, relative frequencies and contingency tables. Fisher’s exact test was used to analyze the association between the categorical variables and also to compare the sensitivity and specificity according to the sampling device. Age differences were analyzed by means of t test. Accuracy was analyzed by calculating the sensitivity, specificity and the area under the receiver operating characteristic (ROC) curve, with the respective confidence intervals. All analysis was carried out using SPSS (version 20) and MedCalc (version 13) softwares.

For the data analysis, the histopathological results of endocervical biopsy/curettage or loop/cone excision were defined as gold standard. Cases were considered positive when the biopsy revealed a histological lesion of CIN2 or worse (CIN2+). Cases considered negative corresponded to biopsies with results that were less severe than CIN2 (<CIN2). Cases in which endocervical biopsy/curettage was not performed were considered negative (<CIN2) when colposcopy was satisfactory and there were no major findings.

## Results

Data from 1,609 patients from the Cancer Prevention Department at the Barretos Cancer Hospital were collected, organized into tables and analyzed. Overall, 1,235 patients were included in the study, 671 of whom were submitted to colposcopy and biopsy (S1 Flowchart).

Most of the study population (n = 1,001; 81%) was between 25 and 64 years of age, the age range approved for screening by the Brazilian health authorities. There was no statistically significant difference in mean age between the groups: Cervex-Brush® Combi (41.2 years) versus Cytobrush+Ayres spatula (42.8 years); p = 0.072 (Student’s t-test). Patients had been referred, irrespective of their age, because of an abnormality in a previous cervical cytology smear. Only women under 18 years of age were excluded from the study.

As the number of samples collected at the Cancer Prevention Department differed slightly from day to day, the total number of tests conducted with one or the other device were different: In 564 patients (45.7%), cytology samples were collected using the Cytobrush+Ayres spatula, while in 671 cases (54.3%), the Cervex-Brush^®^ Combi was used (Table 1).

**Table 1:** Characteristics of the study population (n = 1.235).

Variable	Category	n	%
**Age**	< 25 years	161	13
	25–64 years	1001	81.1
	> 64 years	73	5.9
**Sampling method**	Cervex-Brush® Combi	671	54.3
	Cytobrush+Ayres spatula	564	45.7
**Adequacy of cytology**	Satisfactory	1231	99.7
	Unsatisfactory	4	0.3
**Epithelial representation**	Squamous	241	19.5
	Glandular	3	0.2
	Squamous + glandular	674	54.6
	Squamous + metaplastic	17	1.4
	Glandular + metaplastic	1	0.1
	Squamous + glandular + metaplastic	297	24
	Unknown	2	0.2
**Cytology findings**	No abnormalities	663	53.7
	ASCUS	134	10.9
	ASCH	143	11.5
	AGC	7	0.6
	LSIL	157	12.7
	HSIL	139	11.5
	Invasive carcinoma	6	0.5
	Unknown	2	0.2
**Colposcopic findings**	Inadequate colposcopy	264	21.4
	Normal findings	456	36.9
	Minor abnormalities, suggestive of LSIL	272	22
	Major abnormalities, suggestive of HSIL	220	17.8
	Findings suggestive of invasion	13	1.1
	Not performed	8	0.6
	Miscellaneous with condyloma	2	0.2
**Histology findings**	Cervicitis / No abnormalities	231	34.4
	Condyloma / Atypia associated with HPV	9	1.3
	CIN 1	161	24.1
	CIN 2	92	13.7
	CIN 3	115	17.1
	CIN 2/3	28	4.2
	Carcinoma / Adenocarcinoma	26	3.8
	Undetermined	2	0.3

Four cytology samples (0.3%) were considered unsatisfactory because of low cellularity, and excluded from the study; three collected with Cytobrush+Ayres spatula and one with Cervex-Brush^®^ Combi. In 674 patients (54.6%), the samples had representation of squamous and glandular epithelium, while samples from 301 patients (24.3%) had material from the glandular epithelium, either alone or in conjunction with other epithelia (Table 1). Both devices differed with respect to the endocervical cell representation, with the Cervex-Brush^®^ Combi successfully capturing this type of cell in a greater percentage of cases (82.7%) compared to the Cytobrush+Ayres spatula (74.6%); (p<0.001). The Cervex-Brush^®^ Combi also had a better performance in capturing cells from the transformation zone (84.1 vs. 76.2, p<0.001).

Overall, cervical cytology revealed some type of abnormality in 570 patients (46.1%), and there were also abnormalities in a substantial percentage of colposcopies (n = 507; 41%). According to the biopsy results, a total of 261 cases (38.8%) consisted of CIN2+.

Despite the greater representation of endocervical cells and cells from the transformation zone (glandular and/or metaplastic) achieved with the Cervex-Brush^®^ Combi, there was no statistically significant difference in the cytological diagnosis of each abnormality between the two device groups (Table 2). When we looked at histological and colposcopic findings, there was no significant difference in the proportion of CIN2+ detection according to type of used device (Table 3).

**Table 2:** Distribution of cases according to cervical cell sampling and the different study variables.

Variable	Category	Cervex-Brush® Combi		Cytobrush+Ayres spatula		p-value*
		n	%	n	%	
**Age**	< 25 years	94	14	67	11.9	0.98
	25–64 years	531	79.1	470	83.3	
	> 64 years	46	6.9	27	4.8	
**Adequacy of cytology**	Satisfactory	670	99.9	561	99.5	0.337
	Unsatisfactory	1	0.1	3	0.5	
**Representation of squamous epithelium**	No	1	0.1	3	0.5	0.336
	Yes	670	99.9	559	99.5	
**Representation of glandular epithelium**	No	116	17.3	143	25.4	**<0.001**
	Yes	555	82.7	419	74.6	
**Representation of metaplastic epithelium**	No	505	75.3	412	73.3	0.471
	Yes	166	24.7	150	23.7	
**Representation of the transformation zone** ^**‡**^	No	107	15.9	134	23.8	**< 0.001**
	Yes	564	84.1	428	76.2	
**ASCUS+**	No	366	54.5	297	52.7	0.529
	Yes	305	45.5	267	47.3	
**ASCH+**	No	506	75.4	448	79.4	0.093
	Yes	165	24.6	116	20.6	
**HSIL+**	No	581	86.6	505	89.5	0.115
	Yes	90	13.4	59	10.5	
**Final diagnosis**	< CIN 2	445	75.3	381	76.8	0.569
	CIN 2+	146	24.7	115	23.2	

* Fisher’s exact test.

^‡^ Presence of endocervical or metaplastic cells.

**Table 3:** Distribution of the cases according to the cytological criteria for diagnosis, histological diagnosis and the cervical cell sampling device used.

Cytology criteria	Diagnosis	Cervex-Brush® Combi		Cytobrush+Ayres spatula		p-value*
		n	%	n	%	
**ASCUS+**	< CIN 2	151	54.1	142	58	0.380
	CIN 2 +	128	45.9	103	42	
**ASCH+**	< CIN 2	59	37.3	37	34.3	0.697
	CIN 2 +	99	62.7	71	65.7	
**HSIL+**	< CIN 2	17	19.3	19	32.8	0.079
	CIN 2 +	71	80.7	39	67.2	

* Fisher’s exact test.

Table 4 compares the sensitivity, specificity and area under the ROC curve for each device. Sensitivity was higher for detection of CIN2+ when cytology samples were collected using the Cervex-Brush^®^ Combi (48.6% vs. 33.9%; p = 0.023) and the results of cytology were HSIL+.

**Table 4:** Analysis of the accuracy for a diagnosis of CIN 2+ according to the cytology criteria for diagnosis and the cervical sampling device used.

Cytology Criteria	Indicator of Accuracy	Cervex-Brush® Combi	Cytobrush+Ayres spatula	p-value*
		% (95%CI)	% (95%CI)	
ASCUS+	Sensitivity	87.7 (81.2–92.5)	89.6 (82.5–94.5)	0.699
	Specificity	66.1 (61.5–70.5)	62.7 (57.7–67.6)	0.343
	Area under the ROC curve	0.77 (0.73–0.81)	0.76 (0.72–0.81)	0.797
ASCH+	Sensitivity	67.8 (59.6–75.3)	61.7 (52.2–70.7)	0.186
	Specificity	86.7 (83.2–89.8)	90.3 (86.9–93.1)	0.069
	Area under the ROC curve	0.77 (0.72–0.82)	0.76 (0.70–0.82)	0.734
HSIL+	Sensitivity	48.6 (40.3–57.0)	33.9 (25.4–43.3)	0.023
	Specificity	96.2 (94.0–97.8)	95.0 (92.3–97.0)	0.495
	Area under the ROC curve	0.72 (0.67–0.78)	0.65 (0.58–0.71)	0.063

* Fisher’s exact test.

95%CI: 95% confidence interval.

Gold standard: positive cases (CIN 2+) are those with pathology result of CIN2 or higher. Negative cases (< CIN 2) consist of lesions less severe than CIN 2. In the cases in which endocervical biopsy/curettage was not performed, it was assumed that no disease was present (< CIN 2) when colposcopy was satisfactory and there were no major findings.

## Discussion

In the present study two different parameters of cervical sample collection were assessed: the representation of endocervical cells and the correlation between cytological and histological findings. The evaluation of these parameters in a comparison between two extensively available cervical sampling devices allowed important conclusions to be drawn regarding the best collection device. Bearing in mind that the poor quality of cytology affects the effectiveness of screening programs in developing countries [4, 22, 23], the importance of evaluating parameters capable of improving the quality of the test should be emphasized.

In accordance with the protocol of the Barretos Cancer Hospital, cytology and colposcopy are performed at the same gynecological examination in patients with previous altered Papanicolaou testing. This approach improves the logistics for patients who sometimes have to travel considerable distances to undergo testing. Carrying out cytology sampling and biopsy on the same day prevents the bias of progression or regression of the disease between the time of sample collection for cytology and the time of biopsy [24], thus optimizing analysis. On the other hand, performing colposcopy after cervical cytology sampling may hamper visualization of the cervix if bleeding occurs.

Only a tiny percentage of the samples in this study (0.3%) were considered unsatisfactory for cytological evaluation, what is quite acceptable for liquid-based samples. This methodology reduces the number of unsatisfactory tests due to the fact that fewer collected cells are lost [9, 21, 25], with studies reporting less than 1% of inadequate samples [16]. Previous studies using conventional sampling have reported rates of unsatisfactory cervical cytology samples that range from 2% to 9% with the Cytobrush and 3–15% with the Cervex-Brush^®^ [20]. Furthermore, the standardization of sampling by specialist physicians, in addition to the meticulous preparation and analysis of the slides by an experienced pathology team, contributed to the high rate of satisfactory slides in the present study. In various countries, having more than 10% of slides deemed unsatisfactory is considered indicative of a lack of quality in the cervical sampling or in the preparation of the slides [26].

In the majority of studies, the parameter established to indicate the quality of cervical cytology is the percentage of exams that include endocervical cells [10, 27], since the presence of these cells is a guarantee that the squamocolumnar junction was represented [3]. The possibility of a cytology test having false-negative results is greater when glandular and metaplastic cells are not included in the sample [10].

Various studies conducted to compare the Cervex-Brush^®^ and the Cytobrush+Ayres spatula in conventional cytology have established that the Cytobrush+Ayres spatula combination is more effective for the collection of endocervical cells [21, 28]. It could be speculated that the cells fail to detach effectively from the plastic bristles of the Cervex-Brush^®^ when wiped on the slide; however, the two studies in which this comparison was evaluated using liquid-based cytology also found the Cytobrush+Ayres spatula to be better in this respect [21, 29].

The modification made to the Combi-Brush^®^ intended to represent more endocervical cells [20]. To the best of our knowledge, there are no publications in the literature comparing this new device with the Cytobrush. A better detection rate of cytological abnormalities would be expected for the brush with a better endocervical cell collection; however, the percentage of each abnormality was statistically equivalent for the two devices. Other studies have also reported different rates of endocervical cell representation when comparing two different devices, although the prevalence of cervical abnormalities was similar in both groups [29, 30]. It is our belief that greater sample sizes would be required to identify statistically significant differences between cervical abnormalities as a function of the device’s capacity to recover cells from the transformation zone.

To date, the accuracy of cervical cytology has been evaluated in few developing countries [26]. In agreement with previous studies, the parameter established for normalcy in the present study was a normal histology report or, if biopsy was not performed, normal colposcopic findings [3, 26]. The fluctuating accuracy of cervical cytology, as described previously, can be partially attributed to the different parameters used in pairing cytology results with biopsy findings [3, 31], as well as to the population evaluated [3].

Colposcopy and biopsy are the tests most commonly established as the gold standard in clinical practice, although this practice could be questioned, since both colposcopy and the exact site to be biopsied are examiner-dependent [26, 31, 32]. Colposcopic findings alone are not as good a parameter as biopsy for use as the gold standard, since the inter-examiner variation is greater than that found with histological evaluation [26]. On the other hand, even a not perfect gold standard can be used effectively to compare two tests [33] or, as in the present study, two devices. In general, colposcopy overestimates the abnormality of the cervical smear [26, 34]. The inclusion in the accuracy analysis of 456 patients who did not perform biopsy and whose colposcopic findings were normal was necessary because, in clinical practice, if colposcopy is normal, the patient is not submitted to cervical biopsy. Excluding the patients who were not submitted to biopsy from the study would introduce a selection bias.

In the present study, as expected, the sensitivity decreased and specificity increased with worsening the cytological findings. Tests with poor specificity, when applied to populations in which there is a low prevalence of the disease, result in a high percentage of false-positives and, consequently, a low positive predictive value [35]. Since the percentage of patients with more severe cytological abnormalities was high, the positive correlation with the results of biopsy or colposcopy was consequently higher.

The methodology used in the present study is similar to that used in studies conducted by Germain *et al*.,[15] and by Risberg *et al*., [19] In the former, the authors compared the results of cervical sampling with biopsy in 616 patients and found no differences between the Cytobrush and the Cervex-Brush^®^ in detecting cytological abnormalities for the prediction of histological abnormalities, although the endocervical cell representation was better with the Cytobrush. In the study conducted by Risberg *et al*., there was no difference between the Cytobrush and Cervex-Brush^®^ with respect to endocervical cell collection or the prediction of abnormalities at histology. Since the sample size in the present study was larger than that of previous studies, it was possible to detect a better sensitivity for CIN2+ using Cervex-Brush^®^ Combi when the cytology result was HSIL+. This may be due to the fact that Cervex-Brush^®^ Combi extracted more endocervical cells in the smear than the other device [15, 19].

The qualities of the Cervex-Brush^®^ Combi include a better capacity to obtain samples from the transformation zone and a better sensitivity for predicting CIN2+ in a particular group of women. The present study is in line with the hypothesis that patients with severe cytological abnormality, have abnormal cells prone to be found in samples containing cells from the glandular or metaplastic epithelium.

## Supporting Information

S1 Flowchart Flowchart showing the patients included and excluded from the study. (DOCX)
