# Supplementary material for: Comparison of the Cervex-Brush® Combi and the Cytobrush+Ayres Spatula Combination for Cervical Sampling in Liquid-Based Cytology
Source: PLoS One. 2016 Oct 14;11(10):e0164077. doi: 10.1371/journal.pone.0164077 (PMC5065172; doi:10.1371/journal.pone.0164077)
Supplement: S1 Flowchart — (DOCX) [file pone.0164077.s001.docx]

**S1: Flowchart showing the patients included and excluded from the study.**
